# Exploring underutilization of skilled maternal healthcare in rural Edo, Nigeria: A qualitative study

**DOI:** 10.1371/journal.pone.0272523

**Published:** 2022-08-03

**Authors:** Ogochukwu Udenigwe, Friday E. Okonofua, Lorretta F. C. Ntoimo, Sanni Yaya

**Affiliations:** 1 School of International Development and Global Studies, University of Ottawa, Ottawa, Ontario, Canada; 2 Women’s Health and Action Research Centre, Benin City, Edo State Nigeria, Nigeria; 3 Centre for Excellence in Reproductive Health Innovation, Benin City, Nigeria; 4 Federal University Oye-Ekiti, Oye-Ekiti, Ekiti State, Nigeria; 5 The George Institute for Global Health, Imperial College London, London, United Kingdom; University of Leeds, UNITED KINGDOM

## Abstract

**Introduction:**

Existing studies have acknowledged the underutilization of skilled maternal healthcare services among women in rural Nigeria. Consequently, women in rural areas face a disproportionate risk of poor health outcomes including maternal morbidity and mortality. Addressing the challenge of non-use of skilled maternal healthcare in rural areas necessitates the involvement of multi-stakeholders across different sectors who have vital roles to play in improving maternal health. This study explores the factors contributing to the non-use of maternal healthcare services in rural areas of Edo, Nigeria from the perspectives of community elders and policymakers.

**Methods:**

In this qualitative study, data were collected through 10 community conversations (group discussions) with community elders each consisting of 12 to 21 participants, and six key informant interviews with policymakers in rural areas of Edo State, Nigeria. Participants were purposefully selected. Conversations and interviews occurred in English, Pidgin English and the local language; lasted for an average of 9 minutes; were audio-recorded and transcribed to English. Data were manually coded, and data analysis followed the analytical strategies for qualitative description including an iterative process of inductive and deductive approaches.

**Results:**

Policymakers and community elders attributed the non-use of maternal health services to poor quality of care. Notions of poor quality of care included shortages in skilled healthcare workers, apathy and abusive behaviours from healthcare providers, lack of life-saving equipment, and lack of safe skilled pregnancy care. Non-use was also attributed to women’s complex utilization patterns which involved a combination of different types of healthcare services, including traditional care. Participants also identified affordability and accessibility factors as deterrents to women’s use of skilled maternal healthcare.

**Conclusion:**

The emerging findings on pregnant women’s combined use of different types of care highlight the need to improve the quality, availability, accessibility, and affordability of skilled maternal care for rural women in Nigeria.

## Introduction

Globally, an estimated 295,000 women died during and following pregnancy and childbirth in 2017 [1]. Sub-Saharan Africa (SSA) alone accounted for two-thirds of maternal deaths, a vast majority of which could have been prevented. The Sustainable Development Goal 3 is set to reduce the global maternal mortality ratio (MMR) from 216 deaths per 100,000 live births in 2015 to less than 70 deaths per 100,000 live births by 2030 [2]. Achieving this goal could be difficult if preventable maternal deaths continue to occur. The bulk of avertable pregnancy-related deaths and morbidity arises from non-use of skilled maternal healthcare, therefore maternal mortality alone is not a sufficient indicator of a region’s maternal health [3]. Also relevant are indicators related to access and quality of skilled maternal healthcare, particularly among the poorest of the population. Existing studies have established a relationship between favourable maternal health outcomes and the use of skilled healthcare. In countries where the use of specific services such as antenatal care (ANC), in-facility delivery and skilled birth attendance was 98% and above, the MMR was less than 15 (15 deaths per 100,000 live births) [4]. In contrast, countries with an MMR of 500 or higher saw relatively low use of skilled pregnancy care. More precisely, these countries registered less than 50% coverage of adequate ANC, in-facility delivery, and skilled birth attendance. The latter mirrors the maternal health situation in Nigeria where, in 2017, only 43% of births were assisted by skilled health personnel and only 39% occurred in health facilities [5].

Rural women in Nigeria are underserved and tend to use skilled healthcare services much less than their urban counterparts. For instance, in 2018, only 26% of rural births occurred in a health facility, compared to 61% of urban births [5]. Consequently, rural women face a disproportionate risk of poor health outcomes including maternal mortality and morbidity compared to urban women [6]. Evidence on reasons for non-use of skilled care across SSA are varied and often context specific. These variations highlight the importance of context in determining mechanisms to address factors that impede women’s use of skilled maternal care. For instance, employed women in rural Kenya and rural Ethiopia are not using skilled maternal care services but for different reasons. In Ethiopia, employment status among pregnant women was associated with non-use and underutilization of skilled maternal healthcare services due to time constraints imposed by women’s jobs and household chores [7]. In rural Kenya, women with seasonal employment were not using skilled maternal healthcare because they could not afford it [8]. Beyond individual factors, it is also essential to consider how factors interact at the level of women’s community and around the organization of health care. For instance, in Nigeria, women in underserved areas were not using free skilled maternal care services due to the limited availability of maternal care services [9]. In other instances, women were deterred by different dimensions of quality of care including 1) structural factors such as medical supplies, drugs and basic infrastructure at health facilities; 2) process factors such as respectful care, and 3) outcomes such as the addressing and averting pregnancy complications [10–12]. Therefore, understanding these external factors that influence women’s non-use necessitates the involvement of actors across the different sectors, such as community leaders and policymakers, who have vital roles to play in the organization of maternal healthcare.

Across SSA, there is a growing appreciation of community leaders as advocates for maternal health. Community leaders, who are often men, are key decision-makers and custodians of culture who have also been shown to wield significant influence over behaviour change, particularly relating to health [13, 14]. Successful maternal health projects across SSA have noted that engaging traditional leaders in maternal health discussions is paramount to achieving change [13, 15]. Not only are community leaders helpful in identifying emerging issues in their communities, but they also create a conducive environment that enables women to define their own health needs and take the necessary actions. Similarly, policymakers are key stakeholders in maternal healthcare because they establish the policies and contexts within which health care is provided [16]. Studies have reported on the insufficient interest and commitment on the part of policymakers to implement evidence-based policies [17, 18]. This is because when policymakers are not included in the research process, they are constrained in their capacity to access, synthesize and utilize the available evidence to improve policies. Therefore, in order to design policies that deliver maximum health benefits, policymakers must be part of the discussion in understanding maternal health-seeking behaviour and exploring factors that could improve women’s use of skilled maternal healthcare.

The recent COVID19 pandemic has tested the resilience of health systems and countries’ emergency preparedness and response. Healthcare for women and children is often negatively impacted in these scenarios, it is therefore hypothesized that the COVID19 pandemic will worsen the already poor situation of maternal healthcare utilization in Nigeria [19]. In an effort to understand the challenges of providing essential maternal healthcare services while battling the COVID19 pandemic in Nigeria, a countrywide study is relying on the knowledge and skills of various groups of stakeholders whose insights will provide new direction and policy toward mitigating the negative impact of COIVD19 on an already fragile health system [19]. Such studies are recognising that efforts to address the non-use of skilled maternal healthcare should not occur in isolation of one group of stakeholders, but rather across stakeholders who differ in power, worldviews, and objectives. Previous research in rural Nigeria has leveraged the support of an important group of stakeholders, community leaders, to prioritize maternal health [20], but it is also important that perspectives on maternal health are expanded to include the voices of policymakers. Therefore, the current study explores factors contributing to the non-use of maternal healthcare services in rural areas of Edo, Nigeria from the perspectives of both policymakers and community leaders.

This study extends existing literature on access to and use of skilled pregnancy care services from the perspectives of primary users, women in rural Edo, led by the same research team [21]. Results from this study will be useful in developing policy and programs that prioritize maternal health and also increase women’s access to skilled maternal healthcare in rural Edo, Nigeria.

Our findings were reported based on the Consolidated Criteria for Reporting Qualitative Research (COREQ) (See S1 Appendix).

## Method

### Study design

This study uses qualitative description as a qualitative research approach. Qualitative description allows health researchers to examine a phenomenon from a naturalistic perspective and gives a straight description of a phenomenon [22, 23]. In using this method researchers acknowledge existing knowledge of a phenomenon, are able to be flexible in commitment to a theory during the study design and aim for a low-inference interpretation of study findings with an emphasis on describing participants’ views as close to the data as possible. This study presents information from community conversations and key informant interviews with community elders and policymakers respectively, in rural areas of Edo State, Nigeria.

Key informants are individuals possessing particular knowledge, status and skill in and understanding of a subject matter. They provide information in various ways including formal interviews, informal conversations, manuscripts, artifacts, or other forms [24].

For this study, policymakers were considered to be in the best position to offer insights from a community and systems-level on non-use of maternal health services and proffer solutions [25]. Policymakers are crucial to the functioning of the health system, and they make decisions that influence service delivery and uptake of skilled maternal health care. In this study, face-to-face in-depth interviews were conducted with various policymakers.

Community conversations are interactive processes whereby members of the community are engaged around a common problem and arrive at a solution [26, 27]. One key principle of community conversations is that they create spaces for interaction, change and transfer. This form of qualitative research employs different tools for data collection including conversational dialogues with individuals or reviewing artifacts or group discussions. The common theme in these procedures is an orientation towards problem-solving and the role of the individuals in arriving at solutions. Mainstream approaches to dealing with health issues in African communities have involved assembling people for sensitization sessions or awareness-raising activities [27]. The trade-off has been limited opportunities for dialogue and reflection with and among community members. Community conversations create opportunities for people to discuss health issues away from mainstream social environments thereby rejecting the status quo and enhancing new ways of thinking and questioning [28]. In this study, community conversations occurred as group discussions guided by trained facilitators to support critical thinking and problem solving around the low use of skilled maternal healthcare in their communities. In using this approach for this study, the authors recognize that elders of the various communities possess knowledge and capability to bring about social change individually and collectively around the issues of non-use of maternal health care services.

This qualitative study is a part of a larger project by the Women’s Health Action Research Centre in Nigeria and the University of Ottawa, funded under the Innovating for Maternal and Child Health Africa initiative (a partnership of Global Affairs Canada, Canada’s International Development Research Centre, and Canadian Institutes of Health Research). The larger project is a community-based, multi-site, and multi-disciplinary cluster-randomized trial using a mixed methods approach. Details about the larger project have been reported elsewhere [15, 29].

### Research setting

Nigeria’s current population of 206 million makes it the sixth most populous country in the world [30]. Nigeria’s total fertility rate of 5.11 (live births per woman) is projected to drive a population boom and make Nigeria the second largest country in the world by 2100 with a population of 790 million [31]. Nigeria has 36 states and a Federal Capital Territory within which are a total of 774 local government areas (LGAs). It is grouped into six geopolitical zones namely: North West, North East, North Central, South East, South West and South. About 50% of Nigeria’s population reside in rural areas [32]. This study was conducted in Edo State, one of Nigeria’s 36 States and has a population of approximately 3 million people and a land area of 17, 802 square miles [33]. Specifically, this study was conducted in Esan South East (ESE) and Etsako East (ETE), both of which are local government areas (LGA) of Edo state. They are located in the rural and riverine areas of the State. Each LGA comprises 10 political wards within which are several communities, ESE has 100 communities and a total population of 313,717, and ETE has 42 communities and a total population of 145,996 [29]. These study sites were chosen because preliminary baseline assessments revealed high maternal mortality rates and low use of primary healthcare facilities. Primary healthcare centres (PHCs) are government-funded facilities and constitute the main source of skilled maternal healthcare in the two LGAs. There are 25 PHCs in ESE and 28 in ETE. Esan South East has one general hospital in the local government’s headquarters (Ubiaja) and Etsako East has two general hospitals; one in the local government’s headquarters (Agenebode) and another in nearby Fugar City. They are used in addition to existing PHCs for referral for maternal health services.

### Characteristics of participants

For the community conversations, a total of 151 men elders and 7 women elders aged 50–101 years of age participated in the community conversations. Most of them attained post-primary education, whereas a few had no education. The majority were farmers and artisans. The majority of the participants were Christians, and a few declared no religious affiliation. For the key informant interviews, a total of 6 participants included: one senior official within the State Ministry of Health, one senior official within the State Primary Healthcare Development Agency (SPHCDA), two senior officials responsible for PHCs, with one from ETE and the other from ESE LGAs, two senior Local Government officials, one from ETE and the other from ESE.

### Participant recruitment

#### Community elders

Community elders were purposefully recruited using locally accepted methods of establishing contact [34]. The lead investigators for the project (FO, LN) identified indigenous guides in ETE and ESE who then introduced them to the traditional ruler of the communities. The project leaders met with the traditional rulers, explained the purpose of the study, and obtained consent to conduct research with community elders. Community elders within a traditional age-based hierarchy are considered influential and agents of change [35]. Target participants were community elders who were often over 50 years old and who were recognized as influential among their communities. Consultations with the traditional ruler identified 10 communities with traditional age-based hierarchies across the two local governments. Traditional rulers in the communities worked with the research team to schedule meetings and communicated with the elders who gathered for the various group discussions.

#### Policymakers

The lead investigators (FO, LN) identified key informants among known policymakers who held various policy-related positions at the State and Local government levels. Using a purposeful criterion sampling technique [36], participants’ eligibility to participate were determined based on the following criteria; 1) participants were in key policy positions 2) participants had experience within the PHC system. These criteria were necessary to enhance opportunities to obtain rich insights into the non-use of maternal healthcare services in rural areas. The lead investigators contacted each participant via phone or email with information about the study. Face-to-face in-depth interviews were conducted with 6 policymakers from different institutions in ETE and ESE LGAs and at the state level.

### Data collection

#### Community conversations

A total of 10 community conversations were conducted, six were conducted in ESE and four in ETE between July 26 to August 16, 2017. Community conversations were conducted as group discussions, each consisting of 12 to 21 participants. Groups were small enough for open dialogue and freedom of expression yet large enough to maximize discussions from a diverse group of elders [37]. Conversations occurred outdoors and were conducted in Pidgin English and a few in the local languages (Ishan or Etsako). The lead investigators trained local researchers to conduct community conversations. The local researchers were conversant with the traditions of the community and spoke English, Pidgin English, Ishan and Etsako. The lead investigators developed a community conversation topic guide which was piloted in a neighbouring village with 12 elders aged 50 years and older. The guide was designed to engage elders in problem-solving. In keeping with cultural practices and values that emphasize oral modes of agreement, all participants provided verbal consent to participate in the study. They all subsequently provided written informed consent. Community conversations proceeded as follows: first, the researchers raised awareness about maternal mortality and preventative strategies. Second, they discussed challenges to women’s access to skilled pregnancy care and finally, elders proffered solutions and mentioned specific ways they will take action to address issues. Conversations lasted about 90 minutes and ended when no further issues arose. Resolutions generated from the discussions were itemized and read back to the elders at the end of the discussion. The elders provided feedback where necessary.

#### Key informant interviews

Data collection using a key informant interview guide took place from July 16 to August 30, 2017. The principal investigators provided a three-day training session to research assistants who conducted in-depth interviews with participants. In keeping with a qualitative descriptive study, the interview guide was moderately structured to allow for the free description of opinions and experiences. Trained research assistants ran a pilot test of the guide in a community with similar characteristics as the study location. A total of 6 policymakers participated in the study. Quite frequently and for ambivalent reasons, guidelines adopt a sample size of multiples of 10 for interviews. However, studies recommend that in choosing sample size, researchers focus on what has the best opportunity to reach data saturation as that constitutes the gold standard by which purposeful sample sizes are determined in health research [37, 38]. For in-depth interviews, data saturation can be attained in as little as 6 interviews depending on the diversity of data and the sample population [37]. However, the concept of data saturation is also contested within research designs such as qualitative descriptions that stress the uniqueness of each individual’s experience [39]. The authors acknowledge that information obtained from six participants may never truly reach data saturation, the key, however, was to strive to attain thick and rich data. Based on the diverse policymakers interviewed for this study, the authors believe that the data obtained is detailed, nuanced and intricate. Participants signed a consent form prior to participating in the interviews. The interviews lasted for 45 minutes on average and ended when no further issues arose.

See S2 Appendix for relevant interview questions.

#### Ethical considerations

The ethical clearance needed for the larger study was granted by the National Health Research Ethics Committee (NHREC) on April 18, 2017 (reference number NHREC/01/01/2007–18/04/2017). Participants gave their free and informed consent to be enrolled in the study. Participants provided written informed consent prior to participating in this study. They were also informed that once they chose to participate, they could withdraw at any time or chose not to answer any questions, to which there would be no negative consequences. To ensure confidentiality, all personal information were not included in transcripts and quoted texts.

#### Data analysis

The community conversations and key informant interviews were audio-recorded and transcribed by the paid research assistants who were conversant with the spoken languages. Data were transcribed verbatim if in English or translated if in Pidgin English or the local language. Direct translations (word-by-word) were carried out in order to portray the mentality of the participants and present their message as accurately as possible [40]. In cases where syntactical and grammatical structures precluded literal translations, free translations were used to enhance the readability of a text. The primary author (OU) and corresponding author (SY) analyzed the data independently and checked for consistency during frequent discussions. Data analysis was conducted manually and followed the analytical strategies for qualitative description [22]. After immersing themselves in the data, OU and SY read the data line by line, recorded insights, and proceeded to code the data. Next, coded information was sorted to identify patterns and themes from which similarities and differences were identified and extracted for further consideration and analysis. Similar themes generated sub-categories which gave a more general description of the content. Participants’ views of the non-use of maternal health care services in rural Nigeria are presented in the following overarching themes: quality of care, utilization patterns, affordability, and accessibility.

### Trustworthiness

The rigour of a study lies in the degree of confidence in methods used, data obtained and interpretation of data. Trustworthiness of a study is necessary for establishing confidence based on various criteria including credibility, dependability, confirmability and transferability [41, 42]. To enhance the credibility of the study, the authors used investigator triangulation; the coding process involved two coders working independently to code the data and working collaboratively to generate themes. Furthermore, this study used method triangulation by having different methods of data collection namely, in-depth interviews with policymakers and community conversations with influential community elders. After data collection, the lead investigators conducted member checks by feeding back data to the participants from whom data was obtained. To enhance confirmability, the primary author (OU) provided thick descriptions of participants’ responses, alongside relevant quotes to confirm interpretations. Quotes were also chosen to represent a typical response relative to the theme.

## Results

### Reasons for non-use of skilled maternal health care

#### Quality of care

Several policymakers and community elders recognized the critical role of skilled healthcare in ensuring optimal maternal health, however, they acknowledged that this knowledge alone was not sufficient to persuade women to use services. See S1 Table for a summary representation of participants’ responses. Participants opined that the quality of care, or the lack thereof, was a key reason for non-use of skilled maternal healthcare. Poor quality of care manifested as massive shortages in skilled health professionals, apathy and abusive behaviours from healthcare providers, lack of life-saving equipment and lack of safe skilled pregnancy care. Participants indicated that their rural healthcare facilities were in dire need of qualified staff. Reportedly, there was only one doctor attending to the whole local government area. Policymakers attributed this to a massive brain drain underway in the country and a lack of incentives to attract and retain skilled healthcare staff in rural areas. Shortage of qualified healthcare workers has not only created unhealthy work environments for the healthcare workforce, but has also put women in precarious situations. Participants remarked that in some instances, maternal mortality or morbidity had occurred after unqualified staff attended to obstetric emergencies. In other instances, women did not receive care at all due to the lack of healthcare staff. Examples of poor quality of care in rural healthcare facilities were referred to as follows:

“*A lot of studies have been done to try and find out what the problem is*, *but I can tell you what we know from our end*. *We lack human resources*, *so you can have primary health care facility that has one nurse and 2 community health workers*, *in fact in some places you have just 2 staffs*, *2 community health workers and you will agree with me that it will not be possible to provide 24 hours service*, *so they will share themselves*, *you work in the morning*, *I will work in the evening then the night hours are not covered and many of these deliveries come in the night hours*. *So*, *they [pregnant women] get to the healthcare facility and because there are not enough staff*…*she cannot get service*. *So those things affect why the women prefer not to use PHC*.

*(Policymaker*, *State Ministry of health)*.

“*We [women] don’t use PHC because the people are too harsh and do not treat us well*. *They are not qualified to give us injection*…*I feel that if we have qualified doctors and nurses*, *we [women] will use PHC regularly than to take self-treatment*”

*(Community elder*, *ETE)*.

Policymakers and community elders expressed their frustrations over disrespectful care provided by healthcare staff who were often hostile to patients. Participants unequivocally cited it as a major deterrent to women’s use of skilled care in rural communities. As participants explained, women in the community acquire information related to quality from the experiences of friends and family which consequently influences their use of skilled healthcare facilities. Community elders recalled instances of apathy and lack of commitment from health care workers, they deemed it offensive that nurses would not attend to their duties at health facilities and would instead demand that women come to their private houses to receive treatment. Participants explained:

“*What I want to say my people have said it all*. *I was thinking the health centre was built there for the people alone but when you go there*, *they talk to you anyhow*, *you will not see them on duty rather if there is any treatment*, *they take it home to treat*. *There was a day somebody had an accident*, *and the person was rushed to the health centre*, *the nurse was not there to attend to the person*. *When she came*, *she started talking mannerlessly*, *so that is the more reason people do not patronize them*”

*(Community elder*, *ESE)*.

“*They [pregnant women] say I won’t go for antenatal; I don’t believe in it*. *They believe the hospital is a place where if they go there*, *they [healthcare staff] will not have time to attend to them and they are too harsh*”

*(Policymaker*, *PHC official*, *ESE)*

### Utilization patterns

The unique perspectives of community elders highlighted a remarkable utilization pattern of health services among pregnant women; women combined traditional, faith-based, and skilled maternal healthcare services at different points of their pregnancies for different reasons. Women were reportedly skeptical of the benefits of skilled pregnancy care and less likely to use it adequately if they or someone they know had experienced pregnancy complications while using skilled care. One community elder contemplated the efficacy of skilled healthcare and posed the question *“is traditional medicine better than hospital medication*? *I don’t know”*. This accentuated the skepticism and doubt surrounding skilled maternal healthcare within the rural community. Some community elders themselves advocated that women use a combination of different types of health services to avoid complications during pregnancy. The extended narratives of elders bring these issues to relief:

“*I have to say this*, *sometimes*, *though some women register in the health centre and the traditional care centre*, *they still end up having complications in childbirth… if a woman is pregnant and she decides to register in health centre and she delivers safely*, *she will be encouraged*. *But if she has complications in childbirth though registered in a health centre*, *it will not be a good story*. *So*, *we want more enlightenment on this area because the women we have to discuss this with may ask some questions*.”

*(Community elder*, *ESE)*

“*For me*, *when I got married and my wife was pregnant*, *I registered her in the general hospital*, *and also in a traditional Centre*. *Because my understanding is that there are medications in the hospital and also another type of medication from the traditional*. *Because when it is time for her to get the traditional medications*, *she will get it*, *and when is time for her to get the ones from the hospital she will get them too*. *My advice will be*, *any one whose wife is pregnant should register on both side (traditional care centre and hospital) so that there won’t be complications*.”

*(Community elder*, *ESE)*

However, policymakers expressed concern over the dangers of seeking a combination of health services. They narrated instances where women would patronise skilled health services for fetal ultrasound scans and then patronise traditional birth attendants (TBAs) to interpret the results. The results, they reasoned, are often erroneously interpreted which has caused delays in detecting and addressing pregnancy related complications. Furthermore, narratives of policymakers and community elders contrasted attitudes of TBAs who were perceived as kind, diligent, responsive, and willing to provide care with those of healthcare staff who were perceived as abusive, nonchalant, and sometimes unqualified. Participants opined that women chose to use TBAs or patronized them alongside skilled pregnancy care services because TBAs have an essential role in rural communities. TBAs are non-health professionals who are often older women experienced in providing native care to women throughout pregnancy, childbirth and postpartum. There seemed to be a sense of comfort associated with patronising TBAs as a policymaker explained:

“*You know*, *some of our people when they get used to a particular way of doing things*, *even when other modern ways are available*, *they think it is more comfortable*, *more convenient for them to reach these TBAs but we are still doing our enlightenment to ensure that people embrace the health facility*.”

*(Policymaker*, *ETE)*.

### Affordability

Among policymakers, affordability was viewed as a major reason for non-use of maternal healthcare services. Policymakers framed affordability of maternal healthcare to include the economic cost of transportation to health facilities, cost of healthcare services and cost of drugs. Policymakers acknowledged that there were individual variations in the cost of care but generally felt that the cost of maternal health services was too high for the rural population. They attributed the high cost of skilled health services to inadequate funding for the rural healthcare sector. They opined that women often sought cheaper healthcare from TBAs whose services were more affordable.

“*The cost of maternity care is I think between N15*,*000 to N20*,*000 ($58-$68) for the average Nigerian*, *but for the average mother in rural areas*, *it is a huge amount for our population*”

*(Policymaker*, *PHC official*, *ETE)*

“*Very few [pregnant women] go to the health facilities*. *And even the health care facilities are not adequately armed to cater to these women*. *Most of them will opt to go to traditional birth attendants because they do not have the financial wherewithal to go to these adequate facilities*. *It is not fair and many of them do not have jobs so they would rather go to the TBAs that collect their substance*. *If they are farmers*, *they give TBAs foodstuff and they are happy to take the delivery*. *If they are probably shoemakers*, *whatever trade you do*, *the TBAs will collect those materials instead of the cash that they do not have*. *So*, *they would rather patronize TBAs*”.

*(Policymaker*, *SPHCDA)*.

However, not all participants were persuaded about the impact of cost on women’s use of maternal healthcare services. Community elders generally seemed convinced that the quality of care superseded the cost of care for rural households. Reportedly, notions of quality care which emphasized compassionate care, the ability of healthcare providers and facilities to manage maternal health conditions, and accessible health facilities determined women’s use of health care facilities in rural areas, not cost. Community elders acknowledged that services from skilled health facilities are often expensive, but they generally reasserted the importance of skilled healthcare in averting maternal and newborn deaths. They also commented on the state of the facilities which they pointed out were in a state of ruin as a result of neglect. They highlighted the importance of a hygienic and functional facility. Views on this were expressed in community conversations:

“*The reason is not just because of the charges*. *I have never seen anyone who comes back with good attention and complain that the money is too much and tells other women not to go*. *The reason [for non-use] are the nurses are not always on duty for their primary assignment*”

*(Community elder*, *ESE)*.

“*It is not the money that makes us not go there*, *it is the state of the budling*. *I have not been there in many years*, *I hear that the roof of the building is falling*”

*(Community elder*, *ETE)*.

### Accessibility

Participants indicated that women face significant challenges in reaching and accessing care in rural communities. They acknowledged that in communities without a local healthcare facility, women would need to travel long distances to reach health facilities. Even in communities with a local health facility, distance to facilities was further complicated by poor modes of transportation and poor road infrastructure. As participants indicated, poor accessibility often incurred an added cost in seeking care. These factors combined to hinder women’s use of health facilities. Community elders recalled instances whereby women had gone into labour and given birth to their babies on their way to health facilities. They opined that in communities where health facilities were absent, women would often resort to using TBAs who were physically more accessible. Policymakers acknowledged that there was a need for more health facilities to better serve the needs of rural communities.

“*If we need motorcycle to come out from here*, *it is difficult for us*. *Sometimes*, *if our wives fall into labour at night*, *before we can come out from here to access the health centre at Eguare (next PHC community)*, *it will be very difficult*. *You now see that the actual time a woman would have delivered will now be prolonged because she does not arrive early*. *Sometimes*, *women give birth on their way*.”

*(Community elder*, *ESE)*

“*It is not as if they don’t want to use the PHCs but what I feel is making them reluctant to use PHCs*…*those that really live very far can really find it very difficult to access the PHCs*. *So PHCs really need to spread out more and maybe on a ratio to population*. *Now we have so many people attending just one PHC or being serviced by one PHC*… *so we want many more PHCs*…. *so that many more people will have where to go*…*I think that is what has reduced their usage*.

*(Policymaker*, *LGA Official*, *ETE)*

While the issue of access and distant facilities was highlighted among community elders and policymakers, some policymakers were not cognisant of accessibility issues. They remarked on the easily available and assessable skilled healthcare facilities in the communities. They opined that health centres were at the “doorsteps of women in the communities”. Furthermore, community elders lamented certain health facility policies that limited pregnant women’s access to skilled care. Some facilities required patients to present a registration card prior to accessing care. This was a concern especially during health emergencies when urgent care was needed. A participant recalled an incident:

“*I notice that in some of our hospital here*, *the most important thing to them is the card*, *Do you have a card*? *somebody is dying and you are asking the person do you have a card*?”

*(Community elder*, *ETE)*

### Addressing non-use of skilled maternal health services

Participants believed that if healthcare centres were running effectively, more women would patronise them for antenatal, childbirth and postpartum care. Participants proffered solutions to addressing non-use of skilled maternal health services in their communities. Participants agreed that the responsibility ultimately falls to the government to improve the state of primary health centres which is under the purview of the local government. Policymakers acknowledged that the health sector was underfunded and assumed the responsibility for collaborating with the government to address issues related to non-use of skilled maternal care. They suggested that the bottom line to increasing women’s use of skilled care is increased funding. They called for more funding for health education and raising awareness of the benefits of skilled maternal care. They called for increased funding to better equip health facilities with medical supplies and equipment. They called for increased funding to offer better remuneration to attract and retain healthcare workers. Policymakers who cited barriers of cost routinely mentioned community health insurance and cost-sharing schemes as a means to increase women’s use of healthcare services. They intimated on forthcoming policies by the government to enact health insurance schemes and increase funding for the healthcare sector.

“*if you run PHCs effectively*, *the women will not prefer to go to TBAs*. *They will come to your health facilities and because we have deliveries by skilled attendants*, *we will also reduce the maternal mortality and the perinatal mortality and the morbidity*, *that’s the focus of the government and we are working on getting that seriously working*, *that is*, *our PHCs working effectively*”

*(Policymaker*, *State Ministry of health)*

“*We are also trying to capture those people who are self-employed*, *because they also will need to benefit from health services and also be able to benefit from this program [health insurance scheme] to reduce out of pocket expenditure because that has been a challenge*, *paying for health services*, *it has been a challenge*. *It is part of the reasons why people don’t really patronize orthodox health care centres*, *they prefer to go to quacks and self-medicate and cause a lot of problems*, *so the state health insurance scheme is in the pipeline*, *the state government is working on it*”

*(Policymaker*, *Ministry of Health)*

“*Insurance and co-sharing*. *We like it*. *We have been having community money*, *we use it to help in urgent needs of pregnant women and children*.”

*(Community elder*, *ESE)*

Community members are not leaving the responsibility to government alone. They disclosed that they already had co-sharing schemes within the communities. These schemes have been used to offset the cost of skilled maternal healthcare services for several women in their communities. They however welcomed the idea of health insurance schemes enacted by the government. Furthermore, community elders indicated their willingness to collaborate with the government in establishing a health facility in their government. Some communities offered their land upon which to build a healthcare facility, others offered to provide hands-on assistance in building PHCs.

## Discussion

This study explored perceptions of community elders and policy makers on the non-use of skilled pregnancy care in rural Edo State. While community elders and policymakers shared similar views on the topic, their unique understandings and insights into issues faced by women in their communities differed.

Findings from the perspectives of community elders and policymakers indicate that women were not using skilled pregnancy care for a variety of reasons. Community elders contended that the use of skilled pregnancy care was driven primarily by quality considerations, not cost. This finding was corroborated by a study in Tanzania where pregnant women in rural and predominantly poor communities were shown to patronize private healthcare facilities more than government facilities even though maternity services in private facilities were twice as expensive as at government facilities [43]. The women reported deficiencies in the quality of care in government facilities as reasons for non-use. From participants’ responses, notions of quality revolved around the severe shortage of skilled healthcare staff, lack of respectful care, and inadequate medical supplies, equipment and infrastructure. This is consistent with studies conducted in rural Nigeria that confirmed that the different dimensions of quality include: 1) structural factors such as medical supplies, drugs and basic infrastructure at health facilities; 2) process factors such as respectful care, and 3) outcomes such as the addressing and averting pregnancy complications determine women’s use of skilled health facilities [10–12, 44, 45]. In discussing the quality of care, policymakers emphasized the importance of respectful care in determining women’s use of skilled care. This is consistent with studies in Uganda, where respectful maternal care was shown to be a key determinant in women’s use of maternal care services. Respectful care, conceptualized as communication with healthcare staff was a major concern for mothers who reported that respectful communication with their midwives encouraged their use of skilled pregnancy care, while disrespectful communication such as verbal abuse, discouraged mothers from using skilled care facilities [46]. Furthermore, similar findings across Africa indicate the association between the severity of lack of qualified staff and basic medical supplies and pregnant women’s use of healthcare facilities [47, 48]. Furthermore, while out-of-pocket cost has often been cited as an impediment to women’s use of skilled pregnancy care in rural Nigeria [49–51], the current study saw a contradictory and somewhat unexpected finding. Community elders did not deem the cost of skilled pregnancy care catastrophic, although both elders and policymakers acknowledged it as a barrier. The insights of community elders on cost are not surprising because men in this community are often ascribed the role of financial providers [21]. Furthermore, studies across Africa have highlighted the prevalence of men and community engagement in maternal health [52].

It is important to recognize that in most rural communities, skilled modern healthcare coexists with indigenous healthcare. Rural residents rely on indigenous medicines for a large proportion of their healthcare needs. As indicated by community elders and policymakers, women often combined different types of pregnancy care, sometimes to their detriment. Compared to policy makers who trusted the efficacy of skilled pregnancy care, some community elders questioned its efficacy and disclosed that their beliefs influenced women’s use of skilled pregnancy care. Similar findings in Ethiopia and Kenya corroborate the influence of community leaders over health behaviors in their community [13]. Furthermore, policymakers were aware of widespread beliefs in communities that the different types of health providers specialize in different pregnancy related health conditions and opined that this influenced women’s utilization patterns. Their observations are comparable to similar studies in rural Nigeria where women were shown to have utilization patterns that combined different types of maternal care including formal, traditional and religious pregnancy care [12, 53, 54]. Reasons for this were attributed to women’s attitudes and beliefs, as well as geographic and financial limitations. Other studies across Africa have noted that women’s continued use of TBAs even with the availability of skilled pregnancy care is driven by various contextual factors such as notions of provider expertise, health experiences and outcomes of family members and friends [55]. This finding raises policy implications. TBAs are well established in the community, autonomous of the formal health system and are integral parts of the religious and cultural systems of their community. They use practical approaches and experiential knowledge, steeped in religious and cultural norms and practices to provide care to pregnant women [56]. Their influence can be leveraged to work collaboratively with the formal healthcare system to provide support and health promotion activities during pregnancy and childbirth.

Views from policymakers and community elders on accessibility issues underscores the gaps in the physical coverage of health facilities in their communities, which consequently leaves some members of the community underserved. As participants noted, geographic distance to facilities impedes women’s use of skilled pregnancy care. This is worsened by the limited availability of infrastructure such as good roads and emergency or ambulatory services. Studies in rural Nigeria and rural Ghana showed that distance was a determinant of women’s use of maternal health services [57, 58]. Participants suggest that building more facilities will ensure wider coverage among the community and essentially increase healthcare use. In addition to building facilities, participants recommended improving infrastructure in facilities, availing communities with emergency transportation services, improving medical supplies and equipment. Prior research has identified similar strategies to enhance use of health facilities in rural communities in Nigeria [59]. This prior research also suggests integration and coordination among health providers to provide holistic care during pregnancy, delivery and postpartum. For instance, the use of health cards with relevant health information would enhance access to different health facilities and prompt management of health conditions [59].

It is widely acknowledged that community participation is key to successfully implementing and maintaining health services in rural areas. Community elders offered practical assistance in the form of their community land and labour to support health facilities in their communities. The community was also mobilizing financial resources to aid community members utilize skilled health facilities. These narratives of community support for skilled healthcare are promising. Evidence has shown that skilled health service delivery and uptake are enhanced with active community participation whereby communities have a stake in ensuring quality services are provided and sustained [59]. Health insurance schemes were proposed by policymakers. Likewise, community elders welcomed the idea of a more structured health insurance scheme. Nigeria continues to face challenges in enrollment and uptake of community health insurance [60, 61], however, evaluation of the scheme in Rwanda where more than 90% of the population were covered by health insurance showed improved skilled medical care utilization and protection of households from catastrophic cost of healthcare [61]. Furthermore, recent policy initiatives in Nigeria, as discussed by policymakers, could strengthen maternal health service delivery, and improve women’s use of skilled care. Policy makers mentioned their plans to embark on a cost recovery mechanism for rural public health facilities known as the drug revolving fund. Through this system, revenue generated from the sale of drugs to patients is used to purchase new drugs. The system will ensure sustainability and continuity of essential drugs while also making them affordable for patients [62]. Furthermore, ongoing policy initiatives in Nigeria such as the Primary Health Care Under One Roof (PHCUOR), aims to integrate primary health care under one authority and enhance access to funds for PHCs. Funds obtained through this policy can be allocated towards the provision of essential drugs, the maintenance of healthcare facilities, health care transportation, and the development of human resources for rural PHCs [63, 64].

Triangulation of methods and participants elicited rich and diverse views of reasons for non-use of skilled maternal health services in rural Edo State, however, this study is not without limitations. Majority of the community elders in the study sites are men, therefore the perceptions of men were disproportionately represented. The results from this study could have differed if more data was obtained from women elders who are likely to have lived experience of pregnancy and childbirth. The authors reported and interpreted the data with this in mind. Furthermore, this study did not account for socioeconomic differences between participants as this could impact their perspectives on the affordability of skilled healthcare. However, the authors noted general characteristics of the participants including educational level and sources of employment. Furthermore, it is impossible to rule out perspective bias from policymakers who themselves are responsible for health programs and policies. In analyzing the data, free translations were used to enhance readability of the texts in certain instances, the authors acknowledge that in choosing this method, there is the likelihood of misinterpreting participants’ responses. Findings might not be generalizable to all of rural Nigeria as each community’s priorities and experiences with skilled pregnancy care differ.

## Conclusion

This study explored perceptions of community elders and policy makers on the non-use of skilled maternal healthcare in rural Edo State. Both groups expressed similar knowledge of issues preventing women’s use of skilled care, they also offered unique and sometimes different perspectives on why women do not use skilled maternal healthcare in ETE and ESE. Community elders’ influence on women’s combination of different types of care highlights a need to improve awareness of the importance of skilled pregnancy care at the community level. Findings also highlight the need to strengthen the availability, accessibility, and the affordability of rural healthcare facilities. In addition, the provision of high-quality maternal healthcare should be prioritized. Both policymakers and community leaders deemed disrespectful care a barrier to women’s use of skilled care. It is important that interventions that emphasize patient-centered care be made a priority in order to enhance healthcare staff attitudes. As indicated by the policymakers, substantial funding of the rural healthcare sector is required alongside policy changes that prioritize health systems reforms to address quality issues. It Is also important that pro-poor policies are enacted to reduce the financial burden on the poorest of the population. It is noteworthy that the situation described in this study preceded the COVID19 pandemic which continues to test the resilience of health systems and countries’ emergency preparedness and response especially in underserved communities.

## Supporting information

S1 Appendix Consolidated criteria for reporting qualitative studies (COREQ): 32-item checklist which covers the reporting of studies using interviews and focus groups. (DOCX)

S2 Appendix Key informant interview guide for policy makers. Theme: Defining the problem.
(DOCX)

S3 Appendix PLOS questionnaire on inclusivity in global research. (DOCX)

S1 Table Matrix of themes by participant groups. (DOCX)

## Abbreviations

ANC: Antenatal care
ESE: Esan South East
ETE: Etsako East
KII: Key Informant Interview
LGA: Local Government Area
MMR: Maternal Mortality Rate
PHC: Primary Health Care
TBA: Traditional Birth Attendants
