# Supplementary material for: Exploring underutilization of skilled maternal healthcare in rural Edo, Nigeria: A qualitative study
Source: PLoS One. 2022 Aug 3;17(8):e0272523. doi: 10.1371/journal.pone.0272523 (PMC9348693; doi:10.1371/journal.pone.0272523)
Supplement: S1 Appendix — (DOCX) [file pone.0272523.s001.docx]

**Consolidated criteria for reporting qualitative studies (COREQ): 32-item checklist:**

**Exploring underutilization of skilled maternal healthcare in rural Edo, Nigeria: A qualitative study**

| **No. Item** | **Guide questions/description** | **Reported on Page #** |
| --- | --- | --- |
| **Domain 1: Research team and reﬂexivity** |  |  |
| *Personal Characteristics* |  |  |
| 1. Interviewer/facilitator | **Which author/s conducted the interview or focus group?**  Friday E Okonofua, Lorretta FC Ntoimo coordinated and directed interviews | Methods, page 5 |
| 2. Credentials | **What were the researcher’s credentials? E.g. PhD, MD**  The authors’ credentials are as follows:  - Ogochukwu Udenigwe, MSc  -Friday E Okonofua, MD, PhD  -Lorretta FC Ntoimo, PhD  -Wilson Imongan, MBBS  -Brian Igboin, MSc  -Sanni Yaya, PhD | N/A |
| 3. Occupation | **What was their occupation at the time of the study?**  OU: Doctoral student  FO: Professor  LN: Lecturer  WI: Medical practitioner  BI: Program officer  SY: Professor | N/A |
| 4. Gender | **Was the researcher male or female?**  The authors’ identified genders are as follows:  OU: Female  FO: Male  LN: Female  WI: Male  BI: Male  SY: Male | N/A |
| 5. Experience and training | **What experience or training did the researcher have?**  - OU: quantitative and qualitative research training and experience in qualitative research.  FO: quantitative and qualitative training and extensive experience in maternal and child health, including sexual and reproductive health care.  LN: qualitative and quantitative data analyst, and project coordinator in maternal, child, adolescent health, and family research projects.  WI: extensive experience in reproductive health and primary health care.  BI: skilled in strategic planning, work plan development, budgeting, proposal writing, data management, quantitative and qualitative research data analysis using statistical softwares.  - SY: quantitative and qualitative training and extensive experience in global maternal and child health, including sexual and reproductive health care. | N/A |
| *Relationship with participants* |  |  |
| 6. Relationship established | **Was a relationship established prior to study commencement?**  The lead investigators for the project (FO, LN) had established rapport with the community through scoping studies prior to the study commencement. | N/A |
| 7. Participant knowledge of the interviewer | **What did the participants know about the researcher? e.g. personal goals, reasons for doing the research.**  The study objectives were disclosed to participants as part of the informed consent procedure. | Methods, page 5-8 |
| 8. Interviewer characteristics | **What characteristics were reported about the interviewer/facilitator? e.g. Bias, assumptions, reasons and interests in the research topic**  Trained research assistants informed participants of their affiliation with the Women’s Health and Action Research Centre (WHARC). Trained research assistants were conversant in other languages spoken by participants such as Pidgin English. |  |
| **Domain 2: study design** |  |  |
| *Theoretical framework* |  |  |
| 9. Methodological orientation and Theory | **What methodological orientation was stated to underpin the study? e.g. grounded theory, discourse analysis, ethnography, phenomenology, content analysis**  This study uses qualitative description as a qualitative research approach. Qualitative description allows health researchers examine a phenomenon from a naturalistic perspective and gives a straight description of a phenomenon. In using this method researchers acknowledge existing knowledge of a phenomenon and aim for a low-inference interpretation of study findings with an emphasis on describing participants’ views as close to the data as possible. | Methods, page 5 |
| *Participant selection* |  |  |
| 10. Sampling | **How were participants selected? e.g. purposive, convenience, consecutive, snowball**  The lead investigators for the project identified and purposefully recruited community elders who were over 50 years old and who were recognised as influential among their communities.  The lead investigators also identified key informants among known policymakers who held various policy-related positions at the State and Local government levels. Using a purposeful criterion sampling technique, participants’ eligibility to participate were determined based on the following criteria: 1) participants were in key policy positions 2) participants had experience within the PHC system. E | Methods, page 7 |
| 11. Method of approach | **How were participants approached? e.g. face-to-face, telephone, mail, email**  Community elders were purposefully recruited using locally accepted methods of establishing contact, face to face.  Policy makers were recruited by email (or phone) with information about the study, voluntary participation, and informed consent. | Methods, page 6 |
| 12. Sample size | **How many participants were in the study?**  A total of 151 men elders and seven women elders between 50 and 100 years old participated in the community conversations.  A total of 6 policymakers participated in the study. | Methods, page 7 |
| 13. Non-participation | **How many people refused to participate or dropped out? Reasons**?  None | N/A |
| *Setting* |  |  |
| 14. Setting of data collection | **Where was the data collected? e.g. home, clinic, workplace**  Data collection took place at different locations depending on participants. For instance, Conversations with elders occurred outdoors in community squares, while key informant interviews (KII)-in depth interviews- with policy makers were conducted in convenient locations for participants such as their offices. | Methods, Page 7 |
| 15. Presence of non-participants | **Was anyone else present besides the participants and researchers?**  No non-participants were present during the group discussions. | N/A |
| 16. Description of sample | **What are the important characteristics of the sample? e.g. demographic data**  One set of participants were community elders. Most of them attained post-primary education, whereas a few had no education. The majority were farmers and artisans. Majority were Christians, and a few declared no religious affiliation.  The other set of participants were policy makers and included: one senior official within the State Ministry of Health, one senior official within the State Primary Healthcare Development Agency (SPHCDA), two senior officials responsible for PHCs, with one from ETE and the other from ESE LGAs, two senior Local Government officials, one from ETE and the other from ESE. | N/A |
| *Data collection* |  |  |
| 17. Interview guide | **Were questions, prompts, guides provided by the authors? Was it pilot tested?**  The lead investigators oversaw the development of a community conversation topic guide which was piloted in a neighbouring village with 12 elders aged 50 years and older. The community conversation guide was designed to engage elders in problem solving.  The lead investigators developed a KII guide and on the last day of training, trained research assistants moderated the pilot of the guide in a community with similar characteristics to the study site. | Methods, page 7 |
| 18. Repeat interviews | **Were repeat inter views carried out? If yes, how many?**  Repeat interviews were not carried out. | N/A |
| 19. Audio/visual recording | **Did the research use audio or visual recording to collect the data?**  Community conversations and KII were audio-recorded after obtaining participants’ permission to record. | Methods, page 8 |
| 20. Field notes | **Were ﬁeld notes made during and/or after the interview or focus group?**  Yes, reflective notes were made by trained research assistants during community conversations and the KII. | Methods, page 8 |
| 21. Duration | **What was the duration of the inter views or focus group?**  Conversations lasted about 90 minutes and ended when no further issues arose. Each in depth interview lasted approximately 45 minutes. | Methods, page 8 |
| 22. Data saturation | **Was data saturation discussed?**  For in-depth interviews, data saturation can be attained in as little as 6 interviews depending on the diversity of data and the sample population, however, the concept of data saturation is also contested within research designs such as qualitative description that stress the uniqueness of each individual’s experience. The authors acknowledge that information obtained from six policy makers may never truly reach data saturation, the key however, was to strive to attain thick and rich data. Based on the diverse policymakers interviewed for this study, the authors believe that the data obtained is detailed, nuanced and intricate. | Methods, page 8 |
| 23. Transcripts returned | **Were transcripts returned to participants for comment and/or correction?**  Resolutions generated from community conversations with elders were itemized and read back to the elders at the end of the discussion. The elders provided feedback where necessary.  Transcripts were not returned to policy makers for comment or correction. | Methods, page 7 |
| **Domain 3: analysis and ﬁndings** |  |  |
| *Data analysis* |  |  |
| 24. Number of data coders | **How many data coders coded the data?**  The authors OU and SY coded the data | Methods, page 8 |
| 25. Description of the coding tree | **Did authors provide a description of the coding tree?**  The transcript was read and coded based on identified similarities and patterns in the data. | Methods, page 7-8 |
| 26. Derivation of themes | **Were themes identiﬁed in advance or derived from the data?**  The primary author (OU) and corresponding author (SY) analysed the data independently and checked for consistency during frequent discussions. Data analysis followed the analytical strategies for qualitative description by Neergaard, Olesen, Andersen, and Sondergaard, 2009. After immersing themselves in the data, OU and SY read the data line by line, recorded insights, and proceeded to code the data. Next, coded information were sorted to identify patterns and themes from which similarities and differences were identified and extracted for further consideration and analysis. Similar themes generated sub-categories which gave a more general description of the content. | Methods, page 8 |
| 27. Software | **What software, if applicable, was used to manage the data?**  No software was used | N/A |
| 28. Participant checking | **Did participants provide feedback on the ﬁndings?**  No, the participants did not provide feedback on the findings | N/A |
| *Reporting* |  |  |
| 29. Quotations presented | **Were participant quotations presented to illustrate the themes/ﬁndings? Was each quotation identiﬁed? e.g. participant number**  Participants’ quotations were presented to illustrate themes and findings. | Results, pages 9-14 |
| 30. Data and ﬁndings consistent | **Was there consistency between the data presented and the ﬁndings?**  Yes. | Results, pages 8-14 |
| 31. Clarity of major themes | **Were major themes clearly presented in the ﬁndings?**  Yes, we organized the findings by major themes. | Results, pages 9-14 |
| 32. Clarity of minor themes | **Is there a description of diverse cases or discussion of minor themes?**  Yes, we discussed minor themes in the manuscript and situated them within the broader literature. | Discussion, pages 14-17 |
