# Supplementary material for: Exploring underutilization of skilled maternal healthcare in rural Edo, Nigeria: A qualitative study
Source: PLoS One. 2022 Aug 3;17(8):e0272523. doi: 10.1371/journal.pone.0272523 (PMC9348693; doi:10.1371/journal.pone.0272523)
Supplement: S2 Appendix — Theme: Defining the problem. (DOCX) [file pone.0272523.s002.docx]

| **KII NUMBER:** | **DATE (DD/MMM/YY):** | **START TIME:** | **END TIME:** | **FACILITATOR INITIALS:** |
| --- | --- | --- | --- | --- |

**[Read to participants]**

Thank you very much Sir/Ma for speaking to me today. My name is ____ and our project is on increasing women’s access to skilled pregnancy care to reduce maternal and perinatal mortality in rural Edo, Nigeria specifically. We are doing this project in collaboration with a team from the University of Ottawa and we are being supported by the International Development Research Centre (IDRC), Canada to undergo the project in two local governments in Edo State.

I have already read through the consent form with you, which describes this study in detail, and you have given us permission to speak with you. As a reminder, you are not required to answer all our questions, and you may choose to skip questions.

Do we have your permission to have this interview audio-recorded to also enable us to transcribe appropriately for our report?

The discussion will last approximately 45 minutes. There are no right or wrong answers – just your own experience and thoughts.

Do you have any questions before we begin the discussion?

**Defining the Problem**

1. In your opinion, where do pregnant women in rural parts of Edo State seek antenatal, childbirth and postnatal care services?
2. Can you please tell us why pregnant women may not use primary health care facilities for maternal health care?
3. How would you describe the quality of care provided to pregnant women in the most remote part of Edo state?
4. Can you comment on the challenges that women face in accessing primary health care facilities for pregnancy care?
5. What is your perception of the state of primary health care facilities in Edo state?
6. You have raised various issues and challenges. What is your take on how these issues can be addressed?
