# Supplementary material for: Exploring underutilization of skilled maternal healthcare in rural Edo, Nigeria: A qualitative study
Source: PLoS One. 2022 Aug 3;17(8):e0272523. doi: 10.1371/journal.pone.0272523 (PMC9348693; doi:10.1371/journal.pone.0272523)
Supplement: S1 Table — (DOCX) [file pone.0272523.s004.docx]

| **S3 Table : Matrix of themes by participant groups** | | | | |
| --- | --- | --- | --- | --- |
| **Theme**  **Group** | **Quality of Care** | **Utilization Pattern** | **Affordability** | **Accessibility** |
| **Community Elders** | What I want to say my people have said it all. I was thinking the health centre was built there for the people alone but when you go there, they talk to you anyhow, you will not see them on duty rather if there is any treatment, they take it home to treat. There was a day somebody had an accident, and the person was rushed to the health centre, the nurse was not there to attend to the person. When she came, she started talking mannerlessly, so that is the more reason people do not patronize them. (Elder)  We don’t use PHC because the people are too harsh and do not treat us well. They are not qualified to give us injections…I feel that if we have qualified doctors and nurses, we will use PHC regularly than to take self-treatment (Elder) | I have to say this, sometimes, though some women register in the health centre and in the traditional care centre, they still end up having complications in childbirth… if a woman is pregnant and she decides to register in the health centre and she delivers safely, she will be encouraged. But if she has complications in childbirth though registered in a health centre, is will not be a good story. So, we want more enlightenment on this area because the women we have to discuss this with may ask some questions (Elder)  For me, when I got married and my wife was pregnant, I registered her in the general hospital, and also in a traditional Centre. Because my understanding is that there are medications in the hospital and also other types of medication from the traditional. Because when it is time for her to get the traditional medications, she will get them, and when it is time for her to get the ones from the hospital she will get them too. My advice will be, any one whose wife is pregnant should register on both sides (traditional care centre and hospital) so that there won’t be complications (Elder) | The reason is not just because of the charges. I have never seen anyone who comes back with good attention and complain that the money is too much and tells other women not to go. The reason [for non-use] are the nurses are not always on duty for their primary assignment (Elder)  It is not the money that makes us women not go there, it is the state of the budling. I have not been there in many years, I hear that the roof of the building is falling (Elder) | “If we need motorcycle to come out from here, it is difficult for us. Sometimes, if our wives fall into labour at night, before we can come out from here to access the health centre at Eguare (next PHC community), it will be very difficult. You now see that the actual time a woman would have delivered will now be prolonged because she does not arrive early. Sometimes, women give birth on their way. (Elder)  “ I notice that in some of our hospital here, the most important thing to them is the card, Do you have a card? somebody is dying and you are asking the person do you have a card? (Elder) |
| **Policy Makers** | A lot of studies have been done to try and find out what the problem is, but I can tell you what we know from our end. We lack human resources, so you can have primary health care facility that has one nurse and 2 community health workers, in fact in some places you have just 2 staffs, 2 community health workers and you will agree with me that it will not be possible to provide 24 hours service, so they will share themselves, you work in the morning, I will work in the evening then the night hours are not covered and many of these deliveries come in the night hours. So, they [pregnant women] get to the healthcare facility and because there are not enough staff…she cannot get service. (State Ministry of Health official) | You know, some of our people when they get used to a particular way of doing things, even when other modern ways are available, they think it is more comfortable, more convenient for them to reach these TBAs but we are still doing our enlightenment activities to ensure that people embrace the health facility. (Local government official)  They [pregnant women] say I won't go for antenatal; I don’t believe in it. They believe a hospital is a place where if they go there, they [healthcare staff] will not have time to attend to them and they are too harsh” (PHC official) | “The cost of maternity care is I think between N15,000 to N20,000 ($58-$68) for the average Nigerian, but for the average mother in rural areas, it is a huge amount for our population (PHC official)  “Very few [pregnant women] go to the health facilities. And even the health care facilities are not adequately armed to cater to these women. Most of them will opt to go to traditional birth attendants because they do not have the financial wherewithal to go to these adequate facilities. It is not fair and many of them do not have jobs so they would rather go to the TBAs that collect their substance. If they are farmers, they give TBAs foodstuff and they are happy to take the delivery. If they are probably shoemakers, whatever trade you do, the TBAs will collect those materials instead of the cash that they do not have. So, they would rather patronize TBAs. (State official) | It is not as if they don't want to use the PHCs but what I feel is making them reluctant to use PHCs…those that really live very far can really find it very difficult to access the PHCs. So PHCs really need to spread out more and maybe on a ratio to population. Now we have so many people attending just one PHC or being serviced by one PHC... so we want many more PHCs....so that many more people will have where to go...I think that is what has reduced their usage (Local government official). |
| **Addressing non-use of skilled maternal health services** | | | | |
| **Policy Makers** | If you run PHCs effectively, the women will not prefer to go to TBAs. They will come to your health facilities and because we have deliveries by skilled attendants, we will also reduce the maternal mortality and the perinatal mortality and the morbidity, that’s the focus of the government and we are working on getting that seriously working, that is, our PHCs working effectively. (State Ministry of health)  We are also trying to capture those people who are self-employed, because they also will need to benefit from health services and also be able to benefit from this program [health insurance scheme] to reduce out of pocket expenditure because that has been a challenge, paying for health services, it has been a challenge. It is part of the reasons why people don’t really patronize orthodox health care centres, they prefer to go to quacks and self-medicate and cause a lot of problems, so the state health insurance scheme is in the pipeline, the state government is working on it. (Ministry of Health) | | | |
| **Community Elder** | Insurance and co-sharing. We like it. We have been having community money, we use it to help in urgent needs of pregnant women and children | | | |
